# The Extract of Camellia Seed Cake Alleviates Metabolic Dysfunction-Associated Steatotic Liver Disease (MASLD) in Mice by Promoting Coenzyme Q Synthesis

**DOI:** 10.3390/nu17061032

**Published:** 2025-03-15

**Authors:** Xinzhi Chen, Bolin Chen, Zhigang Li, Li Ma, Qinhe Zhu, Changwei Liu, Haixiang He, Zhixu Zhang, Chuyi Zhou, Guanying Liu, Yuqiao Zhou, Senwen Deng, Shiyin Guo, Yongzhong Chen

**Affiliations:** 1 Hunan Engineering Research Center of Lotus Deep Processing and Nutritional Health Sciences, Hunan Key Laboratory of Economic Crops Genetic Improvement and Integrated Utilization, School of Life and Health Sciences, Hunan University of Science and Technology, Xiangtan 411201, China; 22020901014@mail.hnust.edu.cn (X.C.); zhuqinhe@mail.hnust.edu.cn (Q.Z.); lcvv666@163.com (C.L.); 2109030130@mail.hnust.edu.cn (C.Z.); 2109030121@mail.hnust.edu.cn (G.L.); 2109010315@mail.hnust.edu.cn (Y.Z.); 2 Hunan Academy of Forestry, Shao Shan South Road, No. 658, Changsha 410004, China; cbl92@njfu.edu.cn (B.C.); lzg@hnlky.cn (Z.L.); supermarry1@hnlky.cn (L.M.); 3 Hunan Xianglian Engineering Technology Research Center, Xiangtan 411201, China; manager112@163.com; 4 College of Horticulture, School of Food Science and Technology, Hunan Agricultural University, Changsha 410128, China; zhangzhixu@hunau.edu.cn

**Keywords:** metabolic dysfunction-associated steatotic liver disease (MASLD), camellia seed cake extract, Coenzyme Q, lipid metabolism, energy metabolism

## Abstract

**Background**: Metabolic dysfunction-associated steatotic liver disease (MASLD) is a prevalent metabolic disorder. Camellia seed cake, a byproduct of oil extraction, contains a variety of bioactive compounds. This study investigated the regulatory effects and underlying mechanisms of camellia seed cake extract (CSCE) using a high-fat diet (HFD)-induced MASLD mouse model. **Methods**: Mice were divided into four groups: normal control (N, standard diet), HFD model (M), HFD-fed mice treated with low-dose CSCE (L), and HFD-fed mice treated with high-dose CSCE (H). CSCE was administered via oral gavage for eight weeks. Body weight, blood lipid levels, liver weight, hepatic lipid accumulation, oxidative stress markers, ATP levels, and the NADH/NAD^+^ ratio were measured. Transcriptomic and lipidomic analyses were performed to identify potential regulatory pathways, and qPCR analysis was conducted to confirm the expression levels of essential genes. **Results**: CSCE significantly reduced HFD-induced increases in body and liver weights, improved blood lipid profiles and hepatic lipid accumulation, alleviated oxidative stress, increased ATP levels, and reduced the NADH/NAD^+^ ratio. Transcriptomic analysis demonstrated notable enrichment of genes associated with oxidative phosphorylation, mitochondrial function, and lipid metabolism after treatment. The lipidomic analysis demonstrated that the hepatic lipid profile of the H group approached that of the N group, with Coenzyme Q9 (CoQ9) and Coenzyme Q10 (CoQ10) levels significantly increased by 173.32% and 202.73%, respectively, compared to the M group. qPCR validation confirmed that CoQ synthesis-related genes (*Coq2–10*, *Pdss1*, *Pdss2*, and *Hmgcr*) were significantly upregulated in the treatment groups. **Conclusions**: CSCE enhances mitochondrial function by promoting CoQ synthesis, alleviates metabolic dysfunction, and could represent a potential natural intervention for MASLD.

## 1. Introduction

Metabolic dysfunction-associated fatty liver disease (MASLD, formerly known as NAFLD [1]), with a global prevalence rate of 30.2%, is an escalating healthcare burden in the world [2]. MASLD embraces a wide range of liver pathologies [3]. The primary characteristics of MASLD include disrupted hepatic lipid metabolism, as well as mitochondrial dysfunction, which are closely linked to metabolic disorders, including obesity, type 2 diabetes, and cardiovascular diseases [4]. Given the complex and multifactorial pathogenesis of MASLD, coupled with the current absence of approved therapeutic agents, the exploration of natural product-based interventions has seen a rise in interest [5].

Fatty acid β-oxidation plays a central role in maintaining hepatic lipid homeostasis. Mitochondrial β-oxidation mainly facilitates the breakdown of short-, medium-, and long-chain fatty acids, while the oxidation of very long-chain fatty acids predominantly occurs in peroxisomes [6]. These two systems work synergistically to sustain the equilibrium of lipid metabolism [7]. However, during the pathological progression of MASLD, metabolic disturbances induced by high-fat diets can significantly suppress mitochondrial β-oxidation, resulting in the abnormal buildup of fatty acid intermediates [8]. This metabolic disruption exacerbates hepatic lipid accumulation, resulting in steatosis and elevated levels of reactive oxygen species (ROS), which trigger oxidative stress, impair mitochondrial function, and worsen hepatocyte metabolic dysfunction [9]. Evidence suggests that enhancing mitochondrial β-oxidation is crucial for alleviating the lipid metabolism disturbances associated with MASLD [10].

Coenzyme Q (CoQ) functions as an electron carrier. It plays a crucial role in the mitochondrial electron transport chain. While CoQ itself does not pump protons, it facilitates electron flow, which is essential for the proton-pumping activities of Complexes I, III, and IV, which generate the proton gradient essential for ATP synthesis [11]. Additionally, CoQ plays a vital role in antioxidant defense through its cycling between its oxidized (ubiquinone) and reduced (ubiquinol), scavenging free radicals, and mitigating mitochondrial damage induced by ROS [12]. Within the context of MASLD, reduced CoQ levels may exacerbate mitochondrial dysfunction, impairing energy metabolism efficiency and promoting excessive lipid accumulation [13]. Although the therapeutic efficacy of CoQ supplementation in MASLD has been established [14,15], significant deficiencies persist in comprehending how natural products could influence endogenous CoQ manufacture to reinstate mitochondrial β-oxidation and lipid equilibrium.

*Camellia oleifera* Abel. is a small tree or shrub indigenous to southern China, classified within the family Theaceae and genus *Camellia*. It is an important oilseed crop [16]. The residue obtained after pressing the oil from camellia oleifera is called seed cake, which is an abundant source of bioactive compounds, including triterpene saponins, flavonoids, and polyphenols. These compounds have demonstrated various therapeutic effects, including anticancer, antioxidant, hypoglycemic properties, and anti-inflammatory [17]. Despite an annual production of 1.97 million tons [18], camellia seed cake remains largely underutilized, often discarded as industrial waste, resulting in resource inefficiency and environmental burdens [19].

We posited that camellia seed cake extract (CSCE) mitigates MASLD by upregulating genes associated with CoQ production, consequently improving mitochondrial electron transport efficiency, boosting β-oxidation, and decreasing lipid accumulation. To test this hypothesis, we evaluated the therapeutic effects of CSCE on MASLD. The regulatory impact of CSCE on metabolic dysfunction was investigated through comprehensive assessments, including transcriptomic, lipidomic, and qPCR analyses.

## 2. Materials and Methods

### 2.1. Preparation of CSCE

The camellia seed cake was provided by the forestry station of the Hunan Academy of Forestry. The seed cake was pulverized and sieved through a 60-mesh sieve. Defatting was performed using petroleum ether with a solid-to-liquid ratio of 1:3 (*w*/*v*). The samples underwent ultrasonic extraction (300 W, 40 kHz, 25 °C, 30 min) followed by centrifugation (25 °C, 2500× *g*, 10 min). The supernatant was removed, and the procedure was repeated four times to ensure thorough defatting. Subsequently, the defatted material was subjected to ultrasonic-assisted extraction using 60% ethanol at 70 °C, with a solid-to-liquid ratio of 1:6 (*w*/*v*), for 60 min. The extract was concentrated via rotary evaporation, and the residue was freeze-dried to get a crude extract powder.

To further purify the extract, 20 g of AB-8 macroporous resin (Tianjin Nankai Hecheng Technology Co., Ltd., Tianjin, China) was accurately weighed and packed into a chromatographic column. The resin was prewashed with ultrapure water at a flow rate of 1 bed volume per hour (BV/h). The crude extract was dissolved in ultrapure water at a concentration of 30 mg/mL, and the pH was adjusted to 3. The prepared solution (0.5 BV) was loaded onto the column at a flow rate of 0.5 BV/h. Following loading, the column was sequentially eluted with 2 BV of ultrapure water and ethanol solutions at gradient concentrations of 20%, 40%, 60%, 80%, and 100% (*v*/*v*) at a flow rate of 1 BV/h. The fraction eluted with 40% ethanol was collected, and ethanol was eliminated through rotary evaporation. The resulting sample was freeze-dried to yield a powdered product. The freeze-dried powdered product was stored at −80 °C in airtight containers until use.

### 2.2. Animal Experiment

In this study, 28 6-week-old male Kunming (KM) mice were purchased from Hunan SJA Laboratory Animal Co., Ltd. (Changsha, China). The average weight was 28 ± 1.2 g before acclimatization feeding. Standard diet (58% carbohydrate, 20% protein, 4% fat, 5% fiber, 10% moisture, 3% mineral, No.: SCXK0006) and high-fat diet (HFD) (90% standard diet and 10% lard *w*/*w*) were provided by Hunan SJA Laboratory Animal Co., Ltd. Mice were housed in autoclaved polycarbonate cages (32 × 22 × 17 cm, one per cage) with poplar wood shavings, which were replaced three times a week. Breeding environmental conditions are: 22 °C temperature and a 12-h light/dark schedule, with continuous availability of food and water. Following a one-week adaptation period on a standard diet, the mice were randomly assigned to four groups (n = 7): the normal control group (N), the HFD model group (M), the low-dose treatment group (L), and the high-dose treatment group (H). Mice in the N and M groups were administered sterile saline via oral gavage, while the L and H groups received daily oral gavage of CSCE at doses of 250 mg/kg and 1000 mg/kg body weight, respectively. Throughout the treatment period, the N group received a standard diet, while the other groups were maintained on an HFD. The current diagnostic criteria for MASLD characterize the condition as the coexistence of hepatic steatosis and metabolic abnormalities [1]. In this study, we determined the successful establishment of the MASLD model based on significantly elevated metabolic parameters such as hepatic steatosis, obesity, blood lipids, and liver lipids compared with the control group. Although MASLD is primarily applied for clinical diagnosis, we believe that this model demonstrates pathological features of metabolic-associated fatty liver disease in mice that are consistent with the current definition of MASLD.

Following an 8-week course of oral treatment, the mice were fasted for 12 h (with continuous availability of water). The mice were anesthetized with sodium pentobarbital at a dosage of 50 mg/kg (i.p.) and euthanized. Blood was collected via retro-orbital bleeding, left to clot at 37 °C for 2–3 h, and then centrifuged (25 °C, 4000× *g*, 10 min) to obtain serum for further analysis. Following blood collection, the liver was excised, washed with saline to remove blood, drained, and weighed. The left lobe of liver tissue was transferred into a sterile EP tube, rapidly frozen in liquid nitrogen, and kept at ultra-low temperatures. Body weight, food intake, liver weight, and liver index were recorded for each mouse.

Animal experiments received approval from the Institutional Animal Protection and Use Committee of Hunan Agricultural University (Ethics Certificate number 2021-2138). The experimental procedures complied with the guide for the Care and Use of Laboratory Animals in the “Regulations for the Administration of Experimental Animals” and “Measures for the Administration of Experimental Animals in Hunan Province”.

### 2.3. Biochemical Analysis

Serum Low-Density Lipoprotein Cholesterol (LDL-C, A113-1-1), High-Density Lipoprotein Cholesterolas (HDL-C, A112-1-1), triglycerides (TG, A110-2-1), and total cholesterol (TC, A111-2-1) were measured. In addition, hepatic levels of TG (A110-2-1), TC (A111-2-1), total protein (A045-2-1), hydrogen peroxide (H_2_O_2_) content (A064-1-1), catalase (CAT) activity (A007-1-1), adenosine triphosphate (ATP) content (A095-1-1), malondialdehyde (MDA) levels (A003-1-2), total superoxide dismutase (T-SOD) activity (A001-1-1), and the ratio of reduced to oxidized nicotinamide adenine dinucleotide (NADH/NAD^+^, A114-1-1) were evaluated using commercially available kits (Nanjing Jiancheng Bioengineering Institute, Nanjing, China). All assays were conducted following the manufacturer’s guidelines. The kit item numbers for each analyte are indicated in parentheses next to each measurement.

### 2.4. Histological Analysis

Liver samples were preserved in paraformaldehyde for two days, thoroughly rinsed, and embedded in paraffin. Sections were dewaxed in xylene for 5 min and rehydrated using a stepwise series of ethanol and water washes. Hematoxylin staining was performed for 5 min, followed by rinsing with water and bluing in a weak alkaline aqueous solution for 30–60 s. The sections were subsequently rinsed with water for 5–10 min. Eosin staining was applied to stain the cytoplasm for 2–5 min, and it was subsequently dehydrated using an ethanol gradient. The sections were cleared with xylene and sealed with neutral resin. Histological alterations in the liver tissues were examined under a Nikon Eclipse E100 microscope (Tokyo, Japan).

### 2.5. RNA Extraction and Transcriptomic Analysis

RNA extraction and sequencing were conducted by Novogene (Beijing, China). Total RNA quantity and integrity were assessed using the RNA Nano 6000 Assay Kit (Agilent Technologies, Santa Clara, CA, USA) on a Bioanalyzer 2100 system, with RNA Integrity Numbers (RINs) > 8.0 being considered acceptable. mRNA was isolated from total RNA using magnetic beads conjugated with poly-T oligonucleotides and subsequently fragmented. cDNA libraries (370–420 bp) were constructed using the AMPure XP system (Beckman Coulter, Beverly, MA, USA) and sequenced on the Illumina NovaSeq 6000 platform. Sequencing data quality was evaluated using Q20 and Q30 values, as well as GC content. Differential expression analysis was performed using DESeq2 (for biological replicates, version 1.20.0) or edgeR (for non-replicated samples, version 3.22.5), with normalization with DESeq or TMM, respectively. The R package version used for RNA-seq analysis was 3.5.0. Genes with |log2(FoldChange)| > 0.5 and *p* < 0.05 were defined as differentially expressed genes (DEGs). Gene Ontology (GO) analysis and Kyoto Encyclopedia of Genes and Genomes (KEGG) pathway analysis were conducted after confirming data quality.

### 2.6. LC-MS/MS Lipidomic Analysis

Lipidomic analysis was conducted by Novogene (Beijing, China). The lipidomic analysis method is the same as that of Zhou et al. [20], except for the mass spectrometry parameters. Our mass spectrometry parameters are Sheath gas pressure: 40 psi; Sweep gas flow rate: 0 L/min; Auxiliary gas flow rate: 10 L/min (negative ion mode: 7 L/min); Spray voltage: 3.5 kV; Capillary temperature: 320 °C; Heater temperature: 350 °C; S-lens RF level: 50; Scan range: 114–1700 *m*/*z*; Automatic gain control (AGC) target (full MS): 3 × 10^6^; Normalized collision energy (NCE): 22, 24, and 28 eV (both positive and negative ion modes); Maximum injection time: 100 ms; Isolation window: 1 *m*/*z*; AGC target (MS/MS): 2 × 10^5^; Dynamic exclusion duration: 6 s.

Raw LC-MS/MS data were analyzed using LipidSearch software (v1.4.16) for peak alignment, feature extraction, and quantification, with lipid species annotated via the LipidSearch database and peak intensities normalized to total spectral intensity. Missing values were processed using Compound Discoverer (CD): metabolites absent in >50% of samples were removed, and remaining gaps were imputed with group medians without applying adjusted *p*-values. Statistical analysis was performed using R v3.4.3 and Python v2.7.6. The multivariate analysis involved data transformation in metaX software (v1.4.16), followed by Principal Component Analysis (PCA) and Partial Least Squares Discriminant Analysis (PLS-DA) to generate Variable Importance in Projection (VIP) scores, with PLS-DA model validity confirmed through 7-fold cross-validation. For univariate analysis, a Tukey test (post hoc test for ANOVA) was used to calculate *p*-values and fold change (FC) between groups. Differential metabolites were selected based on thresholds of VIP > 1, *p*-value < 0.05 (uncorrected), and FC ≥ 1.2 or FC ≤ 0.833. Visualization included volcano plots (R package ggplot2) integrating VIP, log2(FC), and −log10(*p*-value); hierarchical clustering heatmaps (R package Pheatmap) with z-score normalized data; and correlation analysis using Pearson coefficients (R cor()) with statistical significance assessed via cor.mtest() (*p* < 0.05) and visualized via R package corrplot. The delivered results did not include enrichment analysis. Raw data and code are available through Novogene upon request and are compliant with their internal standards.

### 2.7. Quantitative Real-Time PCR

Total RNA was extracted from liver tissue using the BioFast SimplyP Total RNA Extraction Kit (BioFlux, Hangzhou, China). Reverse transcription was conducted with the PrimeScript™ RT Reagent Kit (TaKaRa, Dalian, China), and quantitative PCR (qPCR) was conducted with the TB Green^®^ Premix Ex Taq Kit (TaKaRa, Dalian, China). The relative expression levels of target genes were adjusted to β-actin as the internal reference gene and were calculated using the 2^−ΔΔCT^ method. Table 1 presents the primer sequences [21,22]. (The derivative melting curve of *Coq2*, *Coq3*, *Coq4*, *Coq6* are showed in Figure S1).

### 2.8. Statistical Analysis

Statistical analyses and data visualization were performed using GraphPad Prism 8.0.2 (GraphPad Software, San Diego, CA, USA). For multi-group comparisons, one-way analysis of variance (ANOVA) was used. After determining that significant differences existed between the groups, pairwise comparisons were performed using the Tukey test. Statistical significance was defined as *p* < 0.05, with thresholds for higher significance levels set at *p* < 0.01, *p* < 0.001, and *p* < 0.0001.

## 3. Results

### 3.1. CSCE Improves Body Weight and Blood Lipid Profiles

**Figure 1:**
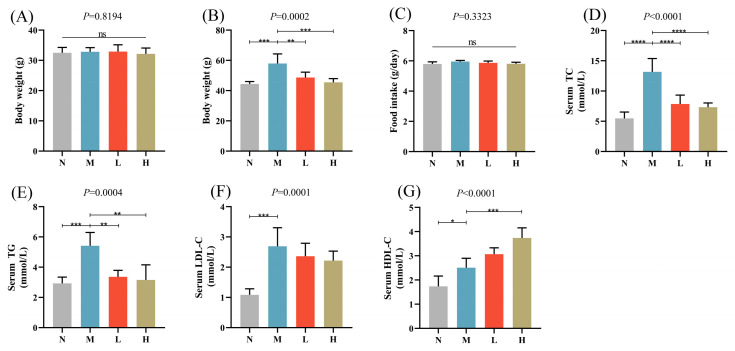
Effects of camellia seed cake extract (CSCE) in Mice fed with high-fat diet (HFD): (**A**) Initial body weight at the start of the experiment, (**B**) Final body weight at the end of the treatment, (**C**) Daily food intake, (**D**) Serum TC levels, (**E**) Serum TG levels, (**F**) Serum LDL-C levels, (**G**) Serum HDL-C levels. * *p* < 0.05, ** *p* < 0.01, *** *p* < 0.001, **** *p* < 0.0001. “ns” indicates no significant difference between groups. The overall *p*-value of the analysis of variance (ANOVA) is marked above each picture.

The therapeutic potential of CSCE for alleviating HFD-induced metabolic disorders was evaluated by administering different doses of CSCE to obese mice over an 8-week period. The impact of CSCE on body weight and blood lipid levels was assessed. At first, no substantial differences were observed in the average body weight among the groups (Figure 1A). However, by the end of the treatment, the body weight of the M group was markedly higher than that of the N group. Conversely, both the L and H groups showed substantially reduced body weights compared to the M group (Figure 1B).

CSCE intervention did not affect food intake, indicating that the observed weight reduction was not due to changes in dietary consumption (Figure 1C). Compared with the N group, the M group exhibited significantly elevated levels of serum TC, TG, and LDL-C, indicating lipid metabolism disorders. Following CSCE treatment, the L and H groups demonstrated significant reductions in TC and TG levels, along with a notable increase in HDL-C levels compared with the M group (Figure 1D–G).

In summary, CSCE effectively mitigated the increases in body weight and improved the blood lipid profiles of HFD-induced obese mice, highlighting its potential as a therapeutic intervention for metabolic disorders.

### 3.2. CSCE Mitigates Hepatic Lipid Accumulation, Oxidative Stress, and Abnormal Energy Metabolism

**Figure 2:**
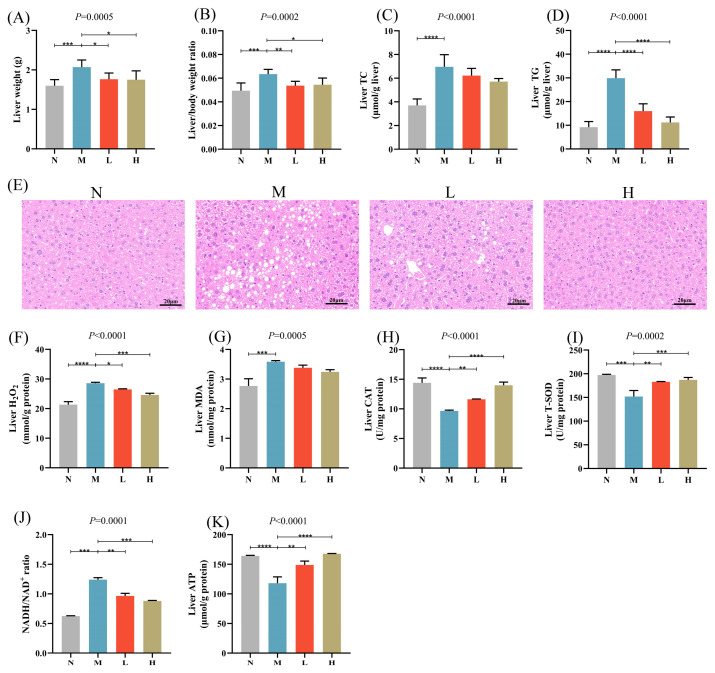
Effects of CSCE on hepatic lipid accumulation, oxidative stress, and energy metabolism in Mice: (**A**) Liver weight, (**B**) Liver index, (**C**) Liver TC levels, (**D**) Liver TG levels, (**E**) Hematoxylin and eosin (H&E) staining of liver tissue. Scale bar: 20 μm, (**F**) Hepatic H_2_O_2_ levels, (**G**) Hepatic MDA levels, (**H**) CAT activity, (**I**) T-SOD activity, (**J**) Hepatic NADH/NAD^+^ ratio, (**K**) Hepatic ATP content. * *p* < 0.05, ** *p* < 0.01, *** *p* < 0.001, **** *p* < 0.0001. The overall *p*-value of the analysis of variance (ANOVA) is marked above each picture.

Hepatic lipid accumulation, oxidative stress, and abnormal energy metabolism were detected in mice fed with HFD, indicating the effective establishment of a MASLD model. Oral administration of CSCE significantly reduced liver weight, liver index, and hepatic TG levels in the L and H groups compared with the M group. There was a reduction in hepatic TC levels, but it was not significant (Figure 2A–D). H&E staining showed pronounced hepatic steatosis in MASLD mice, which was markedly alleviated following CSCE treatment (Figure 2E).

HFD feeding resulted in a significant elevation of hepatic H_2_O_2_ levels, while CSCE administration effectively reduced H_2_O_2_ levels (Figure 2F). Furthermore, CSCE treatment notably decreased hepatic MDA levels and increased CAT and T-SOD activities (Figure 2G–I), indicating a substantial alleviation of hepatic oxidative stress. Specifically, MDA levels were reduced by 5.83% and 9.75% in the L and H groups, respectively, compared with the M group, while CAT activities increased by 20.20% and 44.73%, respectively, and T-SOD activities increased by 20.33% and 23.16%, respectively.

Additionally, CSCE treatment significantly reduced the NADH/NAD^+^ ratio (Figure 2J) and increased hepatic ATP levels (Figure 2K), demonstrating its role in restoring hepatic energy metabolism through enhanced mitochondrial function and oxidative phosphorylation.

### 3.3. Transcriptomic Insights into the Biological Processes and Signaling Pathways Underlying the Ameliorative Effects of CSCE

**Figure 3:**
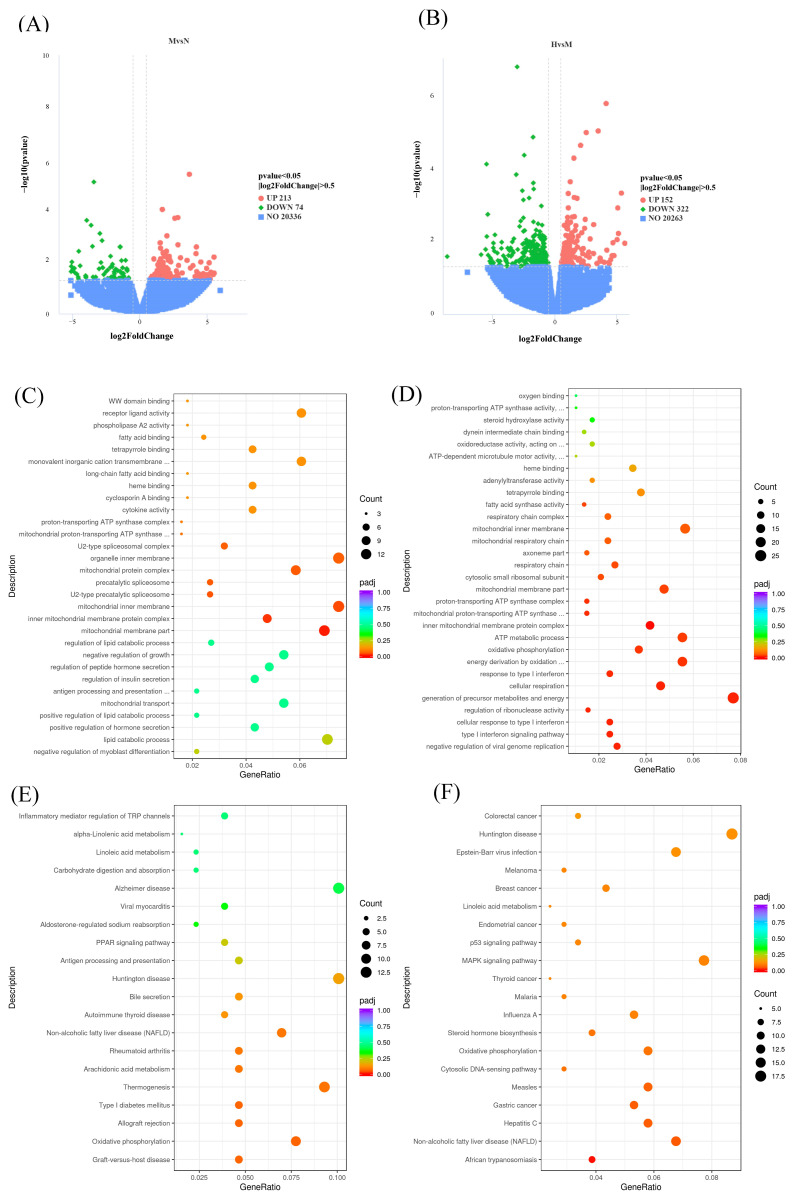
Transcriptomic analysis of CSCE’s effects on metabolic dysregulation in MASLD mice: (**A**) Volcano plot of differentially expressed genes (DEGs) between the N group and the M group, with significance defined as *p* < 0.05 and |log2FoldChange| > 0. Green dots represent downregulated genes, and red dots represent upregulated genes, (**B**) Volcano plot of DEGs between the M group and the H group, with significance defined as *p* < 0.05 and |log2FoldChange| > 0. Green dots represent downregulated genes, and red dots represent upregulated genes, (**C**) GO enrichment bubble chart of DEGs between the N group and the M group, (**D**) GO enrichment bubble chart of DEGs between the M group and the H group, (**E**) KEGG enrichment bubble chart of DEGs between the N group and the M group, (**F**) KEGG enrichment bubble chart of DEGs between the M group and the H group.

To explore the mechanisms by which CSCE mitigates metabolic dysregulation in MASLD mice, we performed RNA sequencing (RNA-seq) to systematically investigate DEGs before and after CSCE administration. Altogether, there were 287 DEGs identified between the N and M groups, with 213 genes upregulated and 74 downregulated in the G group compared to the N group (Figure 3A: specific information is provided in Table S1). Similarly, a total of 474 DEGs were identified between the M and H groups, comprising 152 upregulated and 322 downregulated genes. (Figure 3B, specific information is provided in Table S2).

GO and KEGG enrichment analyses were subsequently performed with respect to the identified DEGs. For the 287 DEGs between the M and N groups, GO enrichment revealed significant associations with terms related to molecular functions, including heme binding and tetrapyrrole binding, suggesting that an HFD disrupts redox homeostasis and exacerbates oxidative stress. In the cellular component category, terms such as mitochondrial inner membrane and associated complexes, including the proton-transporting ATP synthase complex and mitochondrial respiratory chain, were enriched, indicating that HFD may impair mitochondrial structure and electron transport chain function. Biological processes such as lipid catabolic processes, ATP metabolic processes, and oxidative phosphorylation were also enriched, highlighting potential disruptions in lipid and energy metabolism induced by HFD (Figure 3C).

For the 474 DEGs between the M and H groups, GO enrichment revealed significant molecular function terms, including proton-transporting ATP synthase activity and fatty acid synthase activity, suggesting that CSCE treatment may improve mitochondrial function and lipid metabolism by regulating key enzymes. Cellular component terms, such as inner mitochondrial membrane protein complex and mitochondrial respiratory chain complex, were enriched, indicating that CSCE may repair mitochondrial inner membrane function and enhance energy production. Biological processes, including oxidative phosphorylation and energy derivation through the oxidation of organic compounds, were significantly enriched, suggesting that CSCE treatment enhances oxidative phosphorylation efficiency and restores energy metabolism disrupted by HFD (Figure 3D).

KEGG pathway analysis revealed similar findings. For DEGs between the M and N groups, pathways including oxidative phosphorylation, thermogenesis, and MASLD were significantly enriched (Figure 3E). For DEGs between the M and H groups, oxidative phosphorylation and MASLD pathways were predominantly enriched (Figure 3F). These results collectively demonstrate that HFD-induced metabolic dysfunction involves significant disruption of lipid and energy metabolism. CSCE intervention effectively mitigates these abnormalities by modulating pathways associated with mitochondrial function and energy metabolism.

### 3.4. Lipidomic Analysis Reveals Elevated Coenzyme Q Levels with CSCE Treatment

**Figure 4:**
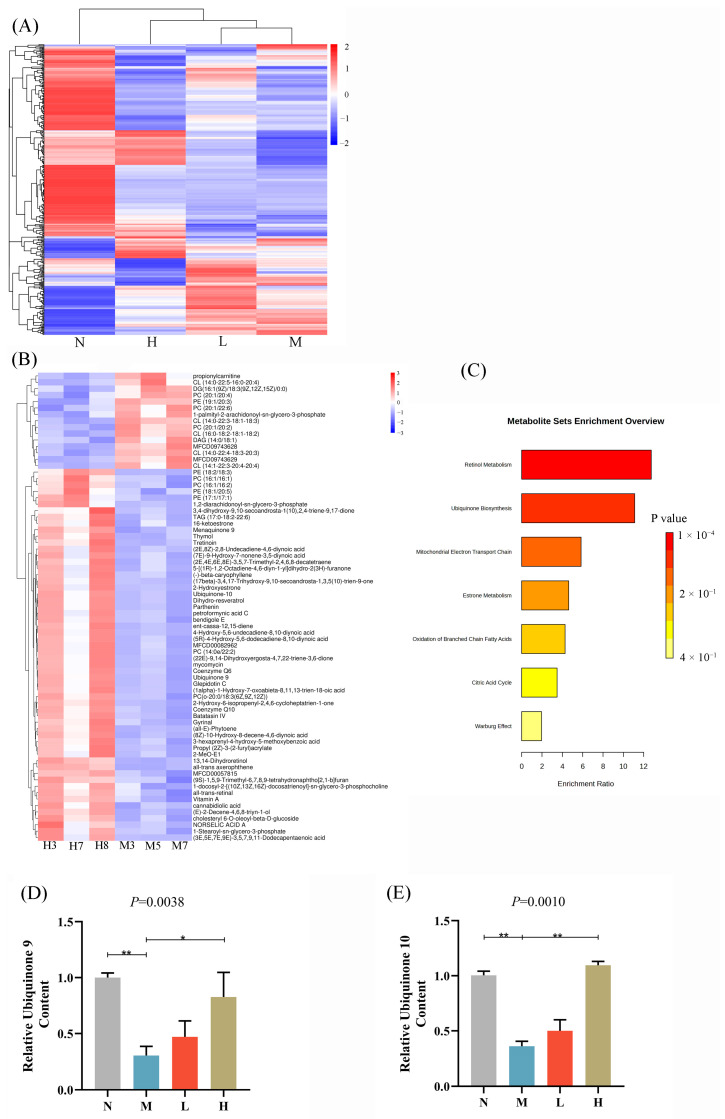
Lipidomic analysis of CSCE’s effects on lipid metabolism in MASLD mice. (**A**) Heatmap visualization of differential lipids across N, M, L, and H groups. (**B**) Heatmap visualization of differential lipids between the H and M groups. The labels in the figure (e.g., H3, H7) are individual mouse numbers. (**C**) KEGG pathway enrichment bar plot of differential lipids between the H and M groups. (**D**) Relative levels of CoQ9 normalized to the N group. (**E**) Relative levels of CoQ10 were normalized to the N group. * *p* < 0.05, ** *p* < 0.01. The overall *p*-value of the analysis of variance (ANOVA) is marked above each picture.

To examine the impacts of CSCE on lipid metabolism in MASLD mice, LC-MS/MS was utilized to obtain lipidomic data from hepatic tissues across all experimental groups. The qualitative and quantitative information of all metabolites can be found in Table S3. The results of the total differential lipids are shown in Table S4. Heatmap visualization revealed contrasting lipid profiles between the N and M groups, indicating significant alterations induced by HFD. Notably, CSCE treatment partially reversed these HFD-induced changes (Figure 4A; specific lipid information is shown in Figure S2). KEGG pathway analysis identified significant enrichment in retinol metabolism pathways in the H group compared with the M group (Figure 4B,C, FDR < 0.05. Figure 4B specific information is provided in Table S5, suggesting a potential regulatory effect of CSCE on this process. The analysis results of differential metabolites for comparisons of other samples are shown in Tables S6–S10. Additionally, trends of enrichment in ubiquinone biosynthesis and mitochondrial electron transport chain pathways were observed, though these did not achieve statistical significance (FDR > 0.05). Despite the lack of statistical significance, these trends align with known metabolic pathways associated with mitochondrial function and energy metabolism.

Quantitative lipid analysis revealed that hepatic levels of CoQ9 and CoQ10 in the M group were significantly reduced by 70.01% and 66.74%, respectively. However, CSCE treatment markedly increased CoQ9 and CoQ10 levels in the H group by 173.32% and 202.73%, respectively, compared to the M group (Figure 4D,E). Notably, the relative levels of CoQ9 and CoQ10 in all groups were calculated based on normalization to the N group as the baseline reference. This pronounced increase in coenzyme Q levels supports the hypothesis that CSCE ameliorates metabolic dysfunction by improving mitochondrial function.

In summary, the lipidomic results demonstrate that CSCE significantly mitigates HFD-induced lipid metabolic dysregulation, potentially through the enhancement of coenzyme Q levels, restoration of mitochondrial function, and promotion of lipid catabolism.

### 3.5. qPCR Analysis of CoQ Biosynthesis-Related Genes

**Figure 5:**
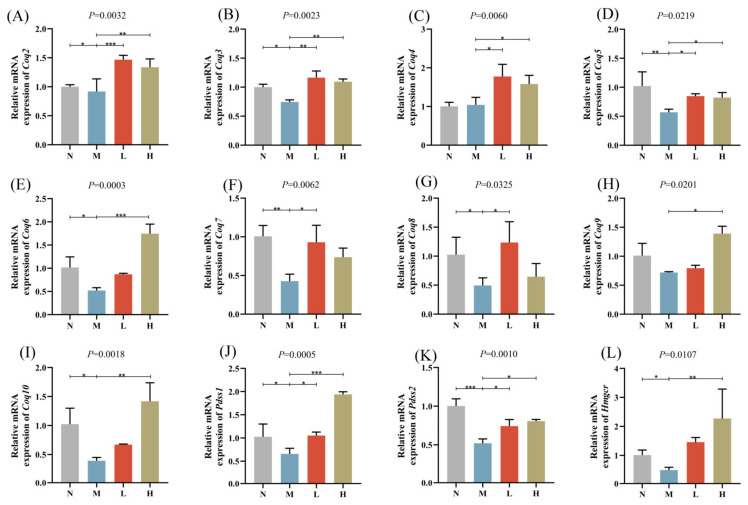
Relative mRNA expression levels of CoQ biosynthesis-related genes: (**A**) *Coq2*, (**B**) *Coq3*, (**C**) *Coq4*, (**D**) *Coq5*, (**E**) *Coq6*, (**F**) *Coq7*, (**G**) *Coq8*, (**H**) *Coq9*, (**I**) *Coq10*, (**J**) *Pdss1*, (**K**) *Pdss2*, (**L**) *Hmgcr*. * *p* < 0.05, ** *p* < 0.01, *** *p* < 0.001.

To validate the findings from transcriptomic and lipidomic analyses, the expression levels of CoQ biosynthesis-related genes were examined using qPCR (Figure 5A–L). The results revealed that CoQ biosynthesis-related genes such as *coenzyme q2,3,5-8,10* (*Coq2, 3, 5-8,10*), 3-hydroxy-3-methylglutaryl-CoA reductase (*Hmgcr*), and decaprenyl diphosphate synthase subunit 1,2 (*Pdss1,2*) were significantly downregulated in the M group compared with the N group. Conversely, the treatment groups (L and H) exhibited a significant upregulation of these genes (*Coq2-10*, *Hmgcr*, *Pdss1*, and *Pdss2*) compared with the M group.

## 4. Discussion

Existing studies have emphasized the potential of camellia seed products to affect glucoregulation and lipometabolism. Our prior research indicated that cold-pressed camellia seed oil mitigated HFD-induced metabolic disorders in rats by activating Adenosine 5′-monophosphate-activated protein kinase and boosting fatty acid oxidation while concurrently suppressing lipid synthesis through the downregulation of sterol regulatory element binding protein-2 and its target genes [23]. Camellia seed cake is abundant in bioactive components, including polysaccharides, flavonoids, and triterpene saponins [24]. With a yearly output of 1.97 million tons [18], the high-value utilization of camellia seed cake remains limited [19]. This study focused on the CSCE, as well as its role and potential mechanisms in modulating mitochondrial function.

A mouse model of MASLD caused by an HFD was established in this work, followed by an 8-week oral gavage treatment with CSCE to evaluate its effects on metabolism disorders. Uncontrolled weight gain is a critical pathogenic factor in MASLD [25]. We observed that the treatment groups of mice administered two different doses of oral CSCE significantly alleviated obesity induced by HFD, with no significant changes in daily food intake, indicating that the weight reduction was not attributed to altered food consumption. Dyslipidemia, characterized by elevated TC, TG, and LDL-C levels, is a common phenotype of MASLD [26]. Our findings demonstrated that CSCE treatment significantly improved the blood lipid profiles of HFD-induced mice. Hepatic lipid accumulation is a hallmark pathological feature of MASLD [27]. The treatment notably reduced liver weight alleviated the liver index and mitigated hepatic TG and TC accumulation. A study by Guo et al. found that camellia seed oil attenuated alcohol-induced liver injury but paradoxically exacerbated hepatic steatosis [28]. In contrast, CSCE effectively reduced lipid accumulation without exacerbating steatosis, suggesting that different components of camellia oleifera may exert distinct effects on liver pathophysiology. Oxidative stress is crucial in the initiation and advancement of MASLD [29]. CSCE treatment reduced oxidative stress markers H_2_O_2_ and MDA levels while enhancing the activities of SOD and GSH-Px. MASLD is associated with mitochondrial dysfunction [30]. CSCE treatment significantly increased hepatic ATP levels and optimized the NADH/NAD^+^ ratio, suggesting that it may restore energy metabolism balance by improving mitochondrial function. These findings indicate the successful establishment of the MASLD model in HFD-fed mice and demonstrate that oral gavage treatment with CSCE effectively ameliorated lipid metabolism disorders.

Disorders of energy metabolism and fatty acid metabolism are major pathogenic factors in MASLD [31]. RNA-seq study indicated that, compared with the N group, the M group demonstrated significant enrichment of DEGs in critical pathways such as oxidative phosphorylation, lipid metabolism, and mitochondrial function. This suggests that a high-fat diet may impair mitochondrial electron transport chain efficiency and fatty acid metabolism balance, leading to energy metabolism dysfunction, lipid accumulation, and exacerbated oxidative stress [32]. Comparative RNA-seq analysis between the H and M groups demonstrated significant enrichment of DEGs in pathways associated with oxidative phosphorylation, lipid metabolism, and mitochondrial function in the H group. GO analysis indicated that these pathways involve functional modules such as proton-transporting ATP synthase activity and mitochondrial respiratory chain complexes. KEGG analysis further identified the enrichment of fatty acid metabolism and oxidative phosphorylation pathways. These findings suggest that the extract may ameliorate HFD-induced metabolic disorders by modulating these key pathways.

The lipidomic analysis further elucidated the mechanism of action of CSCE. Compared with the N group, the hepatic lipid profile of the M group showed significant alterations, characterized by disrupted fatty acid metabolism and impaired mitochondrial function [33]. Lipidomic data from the H group demonstrated a marked regression towards the N group, indicating that CSCE can reverse HFD-induced lipid metabolic disorders and restore lipid homeostasis. KEGG analysis revealed enrichment trends in Coenzyme Q (ubiquinone) biosynthesis and mitochondrial electron transport chain pathways, consistent with transcriptomic findings, supporting the positive regulatory effect of CSCE on mitochondrial function.

CoQ, an essential element of the mitochondrial electron transport chain, serves a crucial function as an electron carrier in oxidative phosphorylation, as well as in ROS scavenging and energy metabolism regulation [34]. This research discovered that hepatic levels of CoQ9 and CoQ10 in the HFD-fed group significantly decreased by 70.01% and 66.74%, respectively, highlighting severe mitochondrial dysfunction caused by HFD. After CSCE treatment, CoQ9 and CoQ10 levels in the H group significantly augmented to 173.32% and 202.73%, respectively, further validating CSCE’s potential in restoring mitochondrial function. This observation strongly supports the hypothesis that CSCE ameliorates metabolic dysfunction by improving mitochondrial function. In mice, CoQ9 is the predominant form of CoQ, serving as a core cofactor in mitochondrial metabolism, whereas CoQ10 is the major form in humans and plays critical roles in various mammalian species [35]. Evaluating both forms provides a comprehensive understanding of CSCE’s regulatory effects on mitochondrial function in mice and lays a theoretical foundation for exploring the potential application of CoQ10 in human metabolic disorders.

CoQ directly determines mitochondrial functional efficiency [34]. The significant increase in CoQ levels observed in this study may enhance mitochondrial electron transport chain efficiency, boosting ATP production and reducing ROS levels. This hypothesis aligns with the upregulation of mitochondrial function-related genes identified in transcriptomic analyses, suggesting that CSCE achieves coordinated regulation of energy and lipid metabolism by improving CoQ levels. Moreover, elevated CoQ levels may support fatty acid β-oxidation, enhancing lipid breakdown efficiency and effectively mitigating hepatic lipid accumulation [15].

Subsequently, we conducted an analysis of the expression levels of CoQ biosynthesis-related genes using qPCR. The biosynthesis of CoQ involves multiple genes, including *Coq2-10*, *Pdss1*, and *Pdss2* [36]. Among these, *Pdss1*, *Pdss2*, and *Coq2* are considered rate-limiting genes in CoQ biosynthesis [37]. Furthermore, CoQ is a lipophilic quinone molecule characterized by a central benzoquinone ring as its core, which is attached to a long hydrophobic side chain composed of several isoprenoid units [38]. The synthesis of CoQ’s side chain depends directly on the mevalonate pathway, with HMGCR serving as the rate-limiting enzyme. HMGCR not only acts as a key regulatory point in cholesterol synthesis but also provides the essential metabolic precursor, isopentenyl pyrophosphate (IPP), for CoQ biosynthesis [39]. HFD negatively impacts CoQ biosynthesis through multiple mechanisms. Firstly, HFD preferentially upregulates cholesterol synthesis via the mevalonate pathway, reducing the availability of isoprenoid precursors for CoQ biosynthesis. Secondly, HFD-induced oxidative stress accelerates CoQ degradation and may inhibit CoQ biosynthetic enzymes, further exacerbating CoQ depletion. Lastly, mitochondrial dysfunction caused by HFD reduces the efficiency of the electron transport chain, thereby reducing the recycling and functional utilization of CoQ. These mechanisms collectively explain the observed reduction in CoQ levels under HFD conditions and underscore the importance of interventions, such as CSCE, that restore CoQ biosynthesis and mitochondrial function [34,40].

Our results revealed that in the M group, the expression levels of *Coq2-10*, *Pdss1*, *Pdss2*, and *Hmgcr* were significantly downregulated compared with the N group. This indicates that HFD may impair mitochondrial function and exacerbate metabolic dysregulation by suppressing the mevalonate pathway and the expression of critical CoQ biosynthesis genes. Conversely, both the treatment groups (L group and H group) exhibited a significantly upregulated expression of these genes, further validating the potential role of the extract in restoring CoQ biosynthesis, improving mitochondrial function, and enhancing energy metabolism.

In summary, CSCE mitigates lipid metabolic disorders induced by HFD through the upregulation of genes associated with CoQ biosynthesis and the enhancement of CoQ production.

Despite uncovering the potential mechanisms of CSCE in ameliorating HFD-induced metabolic dysfunction, several limitations remain to be addressed. While our findings suggest that CoQ biosynthesis is a critical pathway in regulating lipid metabolism, its precise regulatory mechanisms have yet to be fully elucidated. Additionally, the effects and molecular mechanisms of the extract need further verification in in vitro models. Although we identified potential candidate genes through integrated multi-omics data analysis, the specific roles of these key metabolites and genes in the progression of MASLD require further validation through functional experiments such as gene knockout or overexpression studies. Future research ought to concentrate on the key active compounds of the extract and their synergistic effects on tissue-specific metabolism. For instance, recent studies suggest that peptides derived from camellia seed cake exhibit hypoglycemic activity by inhibiting α-glucosidase [41], indicating that CSCE may harbor multifunctional bioactive components beyond lipid metabolism regulation. Further exploration of these compounds—such as polysaccharides, saponins, and peptides—could refine the regulatory mechanisms and provide a basis for clinical translation in metabolic syndromes encompassing both diabetes and MASLD.

## 5. Conclusions

This study systematically investigated the ameliorative effects and potential mechanisms of CSCE on metabolism disorders in HFD-induced mice by integrating animal experiments, transcriptomics, lipidomics, and qPCR analyses. The results demonstrated that CSCE significantly alleviated HFD-induced metabolic dysfunctions, including reductions in body weight and liver weight, improvements in serum lipid profiles (TC, TG, LDL-C, and HDL-C), and decreases in hepatic lipid levels (hepatic TC and TG). Moreover, CSCE mitigated oxidative stress, significantly reduced the NADH/NAD^+^ ratio, and increased hepatic ATP levels, suggesting its potential to improve energy metabolism by enhancing mitochondrial function.

Further transcriptomic and lipidomic analyses, supported by qPCR validation, revealed that CSCE markedly upregulated the expression of CoQ biosynthesis-related genes, leading to increased CoQ levels. The elevated CoQ content not only restored mitochondrial electron transport chain function but also enhanced energy metabolism efficiency, effectively counteracting HFD-induced lipid metabolic disorders. These findings provide important scientific evidence for developing natural product-based interventions targeting metabolic regulation.

## Figures and Tables

**Table 1:** Primer sequences.

Genes	Forward Primer	Reverse Primer
*Coq2*	GCCCACCAGCAGGACAAGAAAGAC	AGCCACAGCAGCGTAGTAGG
*Coq3*	GTGAGCCACCTGGAAATGTT	CCCACGTATGAGTGCTTTT
*Coq4*	GGGGAGACCACAGGATGC	GTCGAGGGTAGACAGCGAGAT
*Coq5*	GGATTCCTTGGGAGGTTCA	GGGCAGTTCTTCAGCGTCT
*Coq6*	CGACGTGGTGGTGTCAGC	AGTTTCTCCAGGGCTTTCTTT
*Coq7*	TGATGGAAGAGGACCCTGAGAAG	GCCTGTATCGTGGTGTTCAAGC
*Coq8*	AGCAAGCCACACAAGCAGATG	CCAGACCTACAGCCAGACCTC
*Coq9*	CCCGAGTTTTCCCGTCC	TGGGCTCCTTCAGCAATG
*Coq10*	TAAACAGAACCCTTCCACCG	CGAAATGCTGATAGTCCTCCA
*Pdss1*	CATCAAAGGACACCAGCAATGT	GCACCACAATAATCGGTCTAAAGG
*Pdss2*	ATGCTGACCTCCAGCCTTTT	GTCACACCTTTGCCAGCTTT
*Hmgcr*	AGCCGAAGCAGCACATGAT	CTTGTGGAATGCCTTGTGATTG
*β-actin*	CGTTGACATCCGTAAAGACC	AACAGTCCGCCTAGAAGCAC

## Data Availability

The data that support the findings of this study are available from the corresponding author upon reasonable request.

## Supplementary Materials

The following supporting information can be downloaded at https://www.mdpi.com/article/10.3390/nu17061032/s1, Figure S1: The derivative melting curves of *Coq2*, *Coq3*, *Coq4*, *Coq6*; Figure S2: Heatmap visualization of differential lipids across N, M, L, and H groups. (Contains detailed lipid information), Table S1: List of differentially expressed genes (M vs. N), Table S2: List of differentially expressed genes (H vs. M), Table S3: The qualitative and quantitative information of all metabolites, Table S4: The list of the total differential lipids, Table S5: List of differential lipids (H vs. M), Table S6: List of differential lipids (M vs. N), Table S7: List of differential lipids (L vs. N), Table S8: List of differential lipids (L vs. M), Table S9: List of differential lipids (L vs. H ), Table S10: List of differential lipid (H vs. N ).
