# Supplementary material for: The Extract of Camellia Seed Cake Alleviates Metabolic Dysfunction-Associated Steatotic Liver Disease (MASLD) in Mice by Promoting Coenzyme Q Synthesis
Source: Nutrients. 2025 Mar 15;17(6):1032. doi: 10.3390/nu17061032 (PMC11944731; doi:10.3390/nu17061032)
Supplement: Supplementary file 1 [file nutrients-17-01032-s001.zip › Notes to tables and figures in the supplementary document.pdf]

Table S1. List of differentially expressed genes (M vs N)

Table Note:

gene_id: gene number

sample: readcount value of each sample after normalization.

group: the mean value of readcount of each group, if the header of the group name is sample.1, it means in order to distinguish it from sample.

log2FoldChange: the ratio of the gene expression level of the treatment group to that of the control group, and then take the logarithm with 2 as the base.

pvalue: p-value for significance test.

padj: p-value corrected for multiple hypothesis testing.

gene_name: gene name

gene_chr: name of the chromosome where the gene is located

gene_start: start position of the gene on the chromosome

gene_end: end position of the gene on the chromosome

gene_strand: information about the positive and negative strands of the chromosome where the gene is located.

gene_length: the length of the gene, the sum of all exon non-overlapping regions from the start to the end of the gene.

gene_biotype: type of gene, e.g. protein coding gene, long chain non-coding gene, etc.

gene_description: description of the gene function.

gene_tf_family: gene transcription factor family annotation

Table S2. List of differentially expressed genes (H vs M)

Table Note:

gene_id: gene number

sample: readcount value of each sample after normalization.

group: the mean value of readcount of each group, if the header of the group name is sample.1, it means in order to distinguish it from sample.

log2FoldChange: the ratio of the gene expression level of the treatment group to that of the control group, and then take the logarithm with 2 as the base.

pvalue: p-value for significance test.

padj: p-value corrected for multiple hypothesis testing.

gene_name: gene name

gene_chr: name of the chromosome where the gene is located

gene_start: start position of the gene on the chromosome

gene_end: end position of the gene on the chromosome

gene_strand: information about the positive and negative strands of the chromosome where the gene is located.

gene_length: the length of the gene, the sum of all exon non-overlapping regions from the start to the end of the gene.

gene_biotype: type of gene, e.g. protein coding gene, long chain non-coding gene, etc.

gene_description: description of the gene function.

gene_tf_family: gene transcription factor family annotation

Table S3. The qualitative and quantitative information of all metabolites

Table Note:

First column: Compound_ID, Metabolite ID (This ID is a randomly assigned number for convenience of retrieval and subsequent analysis, with no actual significance);

Second column: Name, The name of the metabolite (the name of the metabolite in the database);

Third column: Formula, The molecular formula of the metabolite;

Fourth column: Molecular Weight, The relative molecular weight of the metabolite;

Fifth column: RT [min], Retention time;

Sixth column: lipidblast_Results, The matching results of the metabolite with the lipidblast database;

Seventh column: lipidmaps_Results, The matching results of the metabolite with the lipidmaps database.

Eighth column ~: Relative quantitative information of metabolites in each sample (peak area value).

Table S4. The list of the total differential lipids

Table Note:

First column: ID, Metabolite ID;

Second column to the second last column: The mean value of the corresponding metabolites in each sample.

Table S5. List of differential lipids (H vs M)

Table Note:

First column: ID, metabolite ID;

Second column: Name, metabolite description;

Third column: Formula, molecular formula of the metabolite;

Fourth column: Molecular Weight, relative molecular weight of the metabolite;

Fifth column: RT [min], retention time;

Sixth column - penultimate column: quantitative values for different samples;

Seventh column down: FC, comparison of pairwise multiplicity of differences;

Sixth penultimate column: log2FC, log2 value for comparison of pairs of multiplicity of differences;

Penultimate column: Pvalue, comparison of p-values for significance;

Penultimate column: AUC, area of the subject operating characteristic curve;

Penultimate column: VIP, Variable Importance Projection, which reflects the extent to which each sample's quantitative value contributes to the variance.

VIP is usually set to >1;

Penultimate column: Up.Down, upward or downward adjustment;

Penultimate column: Lipid Subclass, the lipid subclass to which the differential metabolite belongs;

Table S6. List of differential lipids (M vs N)

Table Note:

First column: ID, metabolite ID;

Second column: Name, metabolite description;

Third column: Formula, molecular formula of the metabolite;

Fourth column: Molecular Weight, relative molecular weight of the metabolite;

Fifth column: RT [min], retention time;

Sixth column - penultimate column: quantitative values for different samples;
Seventh column down: FC, comparison of pairwise multiplicity of differences;
Sixth penultimate column: log2FC, log2 value for comparison of pairs of multiplicity of differences;
Penultimate column: Pvalue, comparison of p-values for significance;
Penultimate column: AUC, area of the subject operating characteristic curve;
Penultimate column: VIP, Variable Importance Projection, which reflects the extent to which each sample's quantitative value contributes to the variance.
VIP is usually set to >1;
Penultimate column: Up.Down, upward or downward adjustment;
Penultimate column: Lipid Subclass, the lipid subclass to which the differential metabolite belongs;

Table S7. List of differential lipids (L vs N)
Table Note:
First column: ID, metabolite ID;
Second column: Name, metabolite description;
Third column: Formula, molecular formula of the metabolite;
Fourth column: Molecular Weight, relative molecular weight of the metabolite;
Fifth column: RT [min], retention time;
Sixth column - penultimate column: quantitative values for different samples;
Seventh column down: FC, comparison of pairwise multiplicity of differences;
Sixth penultimate column: log2FC, log2 value for comparison of pairs of multiplicity of differences;
Penultimate column: Pvalue, comparison of p-values for significance;
Penultimate column: AUC, area of the subject operating characteristic curve;
Penultimate column: VIP, Variable Importance Projection, which reflects the extent to which each sample's quantitative value contributes to the variance.
VIP is usually set to >1;
Penultimate column: Up.Down, upward or downward adjustment;
Penultimate column: Lipid Subclass, the lipid subclass to which the differential metabolite belongs;

Table S8. List of differential lipids (L vs M)
Table Note:
First column: ID, metabolite ID;
Second column: Name, metabolite description;
Third column: Formula, molecular formula of the metabolite;
Fourth column: Molecular Weight, relative molecular weight of the metabolite;
Fifth column: RT [min], retention time;
Sixth column - penultimate column: quantitative values for different samples;
Seventh column down: FC, comparison of pairwise multiplicity of differences;
Sixth penultimate column: log2FC, log2 value for comparison of pairs of multiplicity of differences;
Penultimate column: Pvalue, comparison of p-values for significance;
Penultimate column: AUC, area of the subject operating characteristic curve;
Penultimate column: VIP, Variable Importance Projection, which reflects the extent to which each sample's quantitative value contributes to the variance.
VIP is usually set to >1;

Penultimate column: Up.Down, upward or downward adjustment;
Penultimate column: Lipid Subclass, the lipid subclass to which the differential metabolite belongs;

Table S9. List of differential lipids (L vs H)

Table Note:
First column: ID, metabolite ID;
Second column: Name, metabolite description;
Third column: Formula, molecular formula of the metabolite;
Fourth column: Molecular Weight, relative molecular weight of the metabolite;
Fifth column: RT [min], retention time;
Sixth column - penultimate column: quantitative values for different samples;
Seventh column down: FC, comparison of pairwise multiplicity of differences;
Sixth penultimate column: log2FC, log2 value for comparison of pairs of multiplicity of differences;
Penultimate column: Pvalue, comparison of p-values for significance;
Penultimate column: AUC, area of the subject operating characteristic curve;
Penultimate column: VIP, Variable Importance Projection, which reflects the extent to which each sample's quantitative value contributes to the variance.
VIP is usually set to >1;
Penultimate column: Up.Down, upward or downward adjustment;
Penultimate column: Lipid Subclass, the lipid subclass to which the differential metabolite belongs;

Table S10. List of differential lipid (H vs N)

Table Note:
First column: ID, metabolite ID;
Second column: Name, metabolite description;
Third column: Formula, molecular formula of the metabolite;
Fourth column: Molecular Weight, relative molecular weight of the metabolite;
Fifth column: RT [min], retention time;
Sixth column - penultimate column: quantitative values for different samples;
Seventh column down: FC, comparison of pairwise multiplicity of differences;
Sixth penultimate column: log2FC, log2 value for comparison of pairs of multiplicity of differences;
Penultimate column: Pvalue, comparison of p-values for significance;
Penultimate column: AUC, area of the subject operating characteristic curve;
Penultimate column: VIP, Variable Importance Projection, which reflects the extent to which each sample's quantitative value contributes to the variance.
VIP is usually set to >1;
Penultimate column: Up.Down, upward or downward adjustment;
Penultimate column: Lipid Subclass, the lipid subclass to which the differential metabolite belongs;

Figure S1. The derivative melting curve of *Coq2*, *Coq3*, *Coq4*, *Coq6*

Figure S2. Heatmap visualization of differential lipids across N, M, L, and H groups. (Contains

detailed lipid information)
