# Supplementary figures and images for: The Extract of Camellia Seed Cake Alleviates Metabolic Dysfunction-Associated Steatotic Liver Disease (MASLD) in Mice by Promoting Coenzyme Q Synthesis
Source: Nutrients. 2025 Mar 15;17(6):1032. doi: 10.3390/nu17061032 (PMC11944731; doi:10.3390/nu17061032)

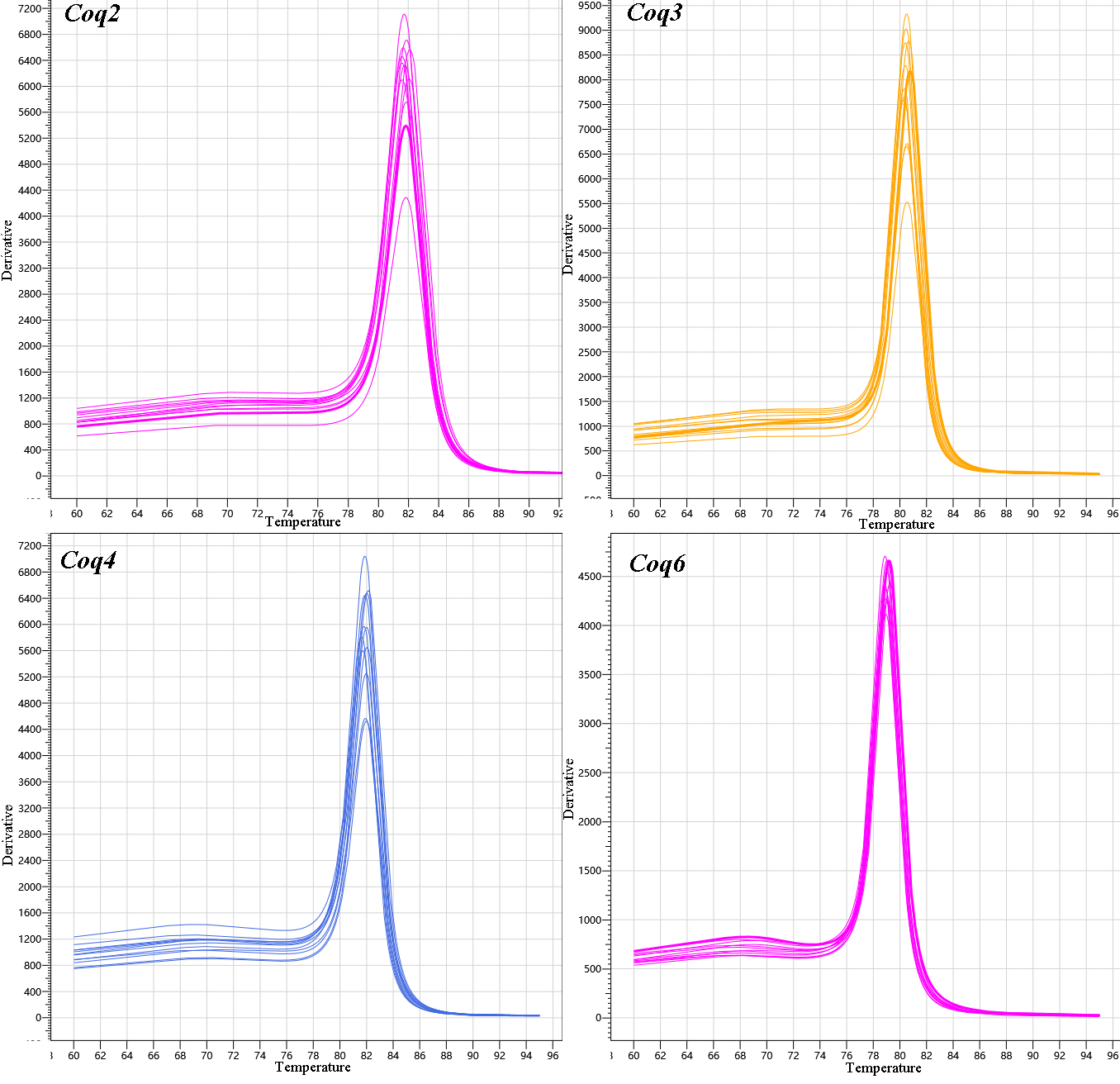

Supplement: Supplementary file 1 [file nutrients-17-01032-s001.zip › Fig S1.jpg]
